# Sensitive plant (*Mimosa pudica*) hiding time depends on individual and state

**DOI:** 10.7717/peerj.3598

**Published:** 2017-07-31

**Authors:** Sarah Reed-Guy, Connor Gehris, Meng Shi, Daniel T. Blumstein

**Affiliations:** Department of Ecology and Evolutionary Biology, University of California, Los Angeles, United States of America

**Keywords:** Sensitive plants, Personality, Optimal escape behavior, Hiding time

## Abstract

The decisions animals make to adjust their antipredator behavior to rapidly changing conditions have been well studied. Inducible defenses in plants are an antipredator behavior that acts on a longer time scale, but sensitive plants, *Mimosa pudica*, have a much more rapid antipredator response; they temporarily close their leaves when touched. The time they remain closed is defined as hiding time. We studied hiding time in sensitive plants and found that individual plants differed significantly in their hiding times. We then showed that the effect of individual explained substantial variation in hiding time on a short time scale. Finally, on a longer time scale, individuality persisted but the amount of variation attributed to individual decreased. We hypothesized that variation in plant condition might explain this change. We therefore manipulated sunlight availability and quantified hiding time. When deprived of light for 6 h, sensitive plants significantly shortened their hiding times. But when only half a plant was deprived of light, hiding times on the deprived half and light exposed half were not significantly different. This suggests that overall condition best explains variation in sensitive plant antipredator behavior. Just like in animals, sensitive plant antipredator behavior is condition dependent, and, just like in animals, a substantial amount of the remaining variation is explained by individual differences between plants. Thus, models designed to predict plasticity in animal behavior may be successfully applied to understand behavior in other organisms, including plants.

## Introduction

Animals use a variety of behavioral and physical defenses to minimize predation risk (Lima & Dill, 1990; Caro, 2005; Verdolin, 2006). Animals reduce the likelihood of predation through constitutive and inducible defenses (Bourdeau & Johansson, 2012; Välimäki, Herczeg & Merilä, 2012). Constitutive defenses do not require environmental stimuli for activation (Harvell, 1990) and will be expressed regardless of predator presence (Clark & Harvell, 1992) while inducible defenses are behavioral responses deployed only in the presence of a threat (Clark & Harvell, 1992). Plants, like animals, also minimize predation risk with inducible defenses (Haukioja, 1991; Adler & Karban, 1994; Dicke & Hilker, 2003). Plants can produce toxins, thorns, and hairs in response to herbivores (Tollrian & Harvell, 1999). When properly used, these inducible defenses confer greater resistance to attack and improve chances of survival (Harvell, 1990). However, the benefits of such defenses are balanced by the cost to growth, reproduction, and survivorship that they incur (Harvell, 1990). Prey should thus behave in ways that optimize the trade-offs between growth and reproduction and defense (Ives & Dobson, 1987). Many defensive phenotypic changes occur on a long time-scale and the trade-offs that they necessitate occur over a plant’s life. While some shorter scale defenses do not involve the production of toxins or structures, they may still be costly to deploy.

Sensitive plants, *Mimosa pudica* (L.), are a tropical shrub and are an ideal system in which to study inducible defenses that occur on a short time-scale. Sensitive plants fold their leaves inward when touched, reducing the surface area exposed to potential predators (Jensen, Dill & Cahill, 2011). This mechanism is initiated when a stimulus causes an efflux of potassium within the leaf, creating a loss of turgor pressure, which closes the leaves and causes them to droop (Allen, 1969; Fleurat-Lessard et al., 1997). Although hiding from predators may reduce herbivory risk, closed leaves do not photosynthesize as effectively (Hoddinott, 1977). Because leaf closure incurs an energetic cost and limits a plant’s ability to photosynthesize, like animals (Cooper, 2003), sensitive plants should be selected to optimize this defensive behavior (Fleurat-Lessard et al., 1997).

Optimal defense is relative to local conditions and individual state (Riechert & Hedrick, 1993; Shudo & Iwasa, 2001). Animals in worse condition prioritize foraging more than conspecifics with ample energy reserves, even at great risk (Bachman, 1993). For instance, yellow-bellied marmots (*Marmota flaviventer*) in worse body condition are predicted to emerge sooner after detecting a predator, accepting greater risk of predation to gain more resources (Rhoades & Blumstein, 2007). Indeed, a meta-analysis found that animals in better condition have longer flight initiation distances because they can afford to flee sooner than their energy-stressed conspecifics and will thus prioritize flight over foraging (Stankowich & Blumstein, 2005).

In sensitive plants, leaf closure reduces photosynthesis by, on average, 40% (Hoddinott, 1977). How an individual plant assesses the need to photosynthesise versus the need to close is dependent on light availability and condition (Simon, Hodson & Roitberg, 2016). Plants more exposed to sunlight will remain closed for longer than plants deprived of sunlight (Jensen, Dill & Cahill, 2011). Furthermore, plants in worse environments will consistently make higher risk light foraging decisions than plants in good environments (Simon, Hodson & Roitberg, 2016). While Jensen, Dill & Cahill (2011) were able to show that light condition modified plant closing behavior, Simon, Hodson & Roitberg (2016) showed that plants were individually consistent in this behavior over the course of several weeks. Importantly, Simon, Hodson & Roitberg (2016) standardized the conditions in the laboratory under which their experiments were carried out to control for genotype and other environmental factors. However, it is poorly understood if and how plants exhibit consistent individual differences in a rapidly changing, non-homogenous field setting.

When conditions are standardized in the lab, animals of the same species, age, and sex may still differ in their behavior and morphology (Carere & Maestripieri, 2013). If these differences vary consistently between individuals, this is evidence of personality or temperament (Dall, Houston & McNamara, 2004). Individual animals that vary along a temperamental continuum have different associated fitness costs and benefits (Dingemanse & Réale, 2005; Carere & Maestripieri, 2013; Sih et al., 2004). Often a consistent temperamental trait is correlated across contexts and this can be described as a behavioral syndrome. For instance, bolder phenotypes may be more adept at mate competition, but when encountering a predator, and if boldness carries over to this context, bolder animals may be more likely to be killed (Smith & Blumstein, 2008). That said, some of the strongest evidence for personality is found under field conditions, as the individual differences remain consistent despite the more variable environments encountered in nature. Just as both animals and plants exhibit antipredator behavior, it is possible that plants, like animals, respond to threats in consistent ways.

We aimed to understand the extent to which sensitive plants are individually specific in their induced hiding response in a natural setting and if the decision to open their leaves to photosynthesize (i.e., forage) are made at a leaf or plant level. The study of individual responses in plants sheds light on how individual plants may respond to environmental challenges. By adopting an individual reaction norm approach, where individual’s responses are studied across multiple contexts and intervals (Stamps & Biro, 2016), we further our understanding of plasticity in plants. If, as seen in animals, the assessment of benefits and costs varies between individual plants, then we expect plants to have individualistic responses. If so, we expect plant condition to explain variation in these responses.

## Materials and Methods

### General methods

Research was conducted under permission of the Government of French Polynesia. We studied sensitive plants near UC Berkeley Gump South Pacific Research Station (17°32′S, 149°50′W) in Mo’orea, French Polynesia between 20 January and 11 February 2016. For our purposes, an individual is defined as a single plant with one root system and at least ten leaves. These plants must have been far enough from other conspecifics to be distinguishable as individuals. The study was conducted at the edge of a wooded area next to a clearing. We assigned numbers to all individuals and elevated stems using metal stakes and twist ties. Plants were allowed to return to their original, expanded positions and acclimate for 48 h before further experimentation. In the following experiments, we touched leaves to induce hiding and measured individual differences in leaf hiding time. Prior to experimentation, all three experimenters standardized the intensity and manner with which they touched the leaf. Trials were conducted between 0700 h and 1600 h. Previous studies have shown that taller plants have increased access to light (Falster & Westoby, 2003). Specific leaf area is also positively correlated with photosynthetic rate (Reich, Ellsworth & Walters, 1998). Thus, we measured plant height (cm), number of leaves per plant, and length of tested leaf (mm) as covariates that we assumed would be associated with plant condition. Plant height (cm) was measured from base of plant, where it joined the ground, to the top of the tallest stem of the plant using a transect tape. Leaf length (mm) was measured using electronic callipers from the base of the leaf where leaflets began to the furthest leaflet tip. Number of leaves was log_10_ transformed to fit a normal distribution.

To explain variation in hiding time in our experiments, we fitted linear mixed effect models using the package lme4 (Bates et al., 2015) on R 3.2.3 (R Core Team, 2015). The parameters used in analysis throughout the study were plant height, leaf number, and leaf length.

For each experiment, we began by fitting a general linear null model with no fixed or random effects. An individual effects only model, one with only individual plant as a random effect, was fitted next. We followed this by fitting a full model, with random and fixed effects. In the experiments with multiple observations of hiding time, we also fitted, a full model where we also estimated random slopes. This model permitted us to estimate the degree to which these individuals differed in their slopes or, in other words, their behavioral response to a repeated stimulus (Dingemanse & Dochtermann, 2013). We compared these models using AIC values to see which best explained the data. When two models were not significantly different, we parsimoniously interpreted the simpler one. We then compared the best model, the one with the lowest AIC, to the null model using a likelihood ratio test. For models with no significant random effects, we then calculated the intra-class correlation coefficient (ICC) as the ratio of variation explained by the individual to total variation. For models with significant random effects, we also calculated adjusted repeatability (Nakagawa & Schielzeth, 2010). Hiding time was log_10_ transformed to fit a normal distribution and we examined residuals with *q*–*q* plots and plotted residuals against fitted values to evaluate model fit and distributional assumptions.

### Experiment 1: is there individual variation in hiding time among sensitive plants?

#### Methods

We aimed to understand the extent to which sensitive plants were individually specific in their induced hiding response, if individuality explained the majority of inter-individual differences in hiding time, and if those differences were significant. We examined variation in hiding time by measuring the hiding times of ten leaves in 14 different individual plants. The environments of these 14 individuals differed somewhat in light exposure and level of disturbance by humans, but we did not formally quantify these microclimatic differences that could explain some differences between individuals but not consistency within individuals. The mean height of the individuals in this experiment was 39.5 cm (±12.4 cm SD, range 22–60.1 cm). The mean number of leaves per plant was 76.3 (±41.4 SD, range 20–157). The mean leaf length was 36.8 mm (±12.9 mm SD, range 4.7–85.5 mm).

#### Results

**Figure 1:**
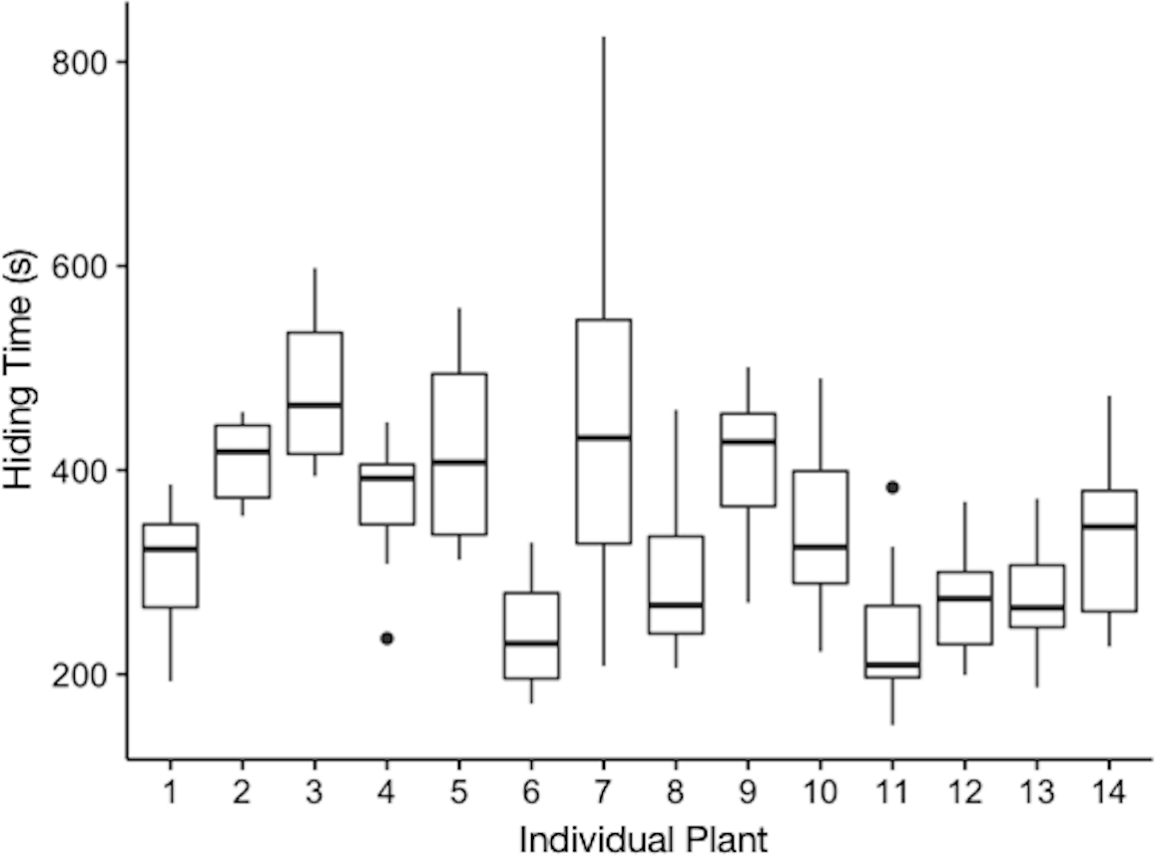
Hiding time (s) as a function of the individual plant. The plotted values are the median hiding times, quartiles, fences (1.5 times the interquartile range), and outliers.

Plants varied in their hiding times (Fig. 1, Table 1). We found that the model only with the random effect of individual plant better explained variation in hiding time than either a null model (*p* < 2.2e^−16^), or a full model with both fixed and random effects. Thus, plants differed in their average hiding time and the intraclass correlation coefficient revealed that 44% of this variation was explained by individual plant. We found that when models included any of the fixed effects, other than leaf length, their ability to predict individual hiding was not improved and these models were therefore excluded from Table 1. The estimate for the effect of leaf length on hiding time was −0.00109 (±0.0009 SEM, *t*-value = − 1.272).

**Table 1:** A comparison of models fitted in R to explain individual variation in leaf hiding time for experiment 1. Data for hiding time were log_10_ transformed.

Description	Model	AIC
Null model	Hiding time ∼ 1	−154
Null model with random effect only	Hiding time ∼1∣ID	−203^a^
Full model	Hiding time ∼ Leaf length + (1∣ID)	−204

**Notes.**

a LRT comparison to null model (*p* < 2.2e^−16^).

### Experiment 2: do individuals vary their hiding time over time?

#### Methods

To assess repeatability under short-term and longer-term time intervals, we measured hiding times in the same leaf of each individual. In experiment 2a, we marked the stem of one leaf on 18 individuals with flagging tape and recorded the hiding time. We then stimulated each leaf again immediately upon fully reopening and did so a total of 4 times. Of 18 individuals, 11 were partially exposed to the sun, seven were fully exposed to the sun, four were on areas that had a history of trimming (not during the duration of this experiment), and 14 were on areas that were not trimmed. In experiment 2b, we marked one leaf on 13 individuals with flagging tape and recorded the hiding time. Each leaf was stimulated every other day at the same time of day as its original trial for a total of four trials. Of 13 individuals, nine were partially exposed to the sun and four were fully exposed to the sun, five were on areas that had a history of trimming (not during this experiment) and eight were on areas that were not trimmed.

In experiments 2a and 2b, we calculated the adjusted repeatability to control for fixed effects in our model (Nakagawa & Schielzeth, 2010). Number of leaves was log_10_ transformed to fit a normal distribution. Again, residuals were plotted to confirm normality.

Experiment 2a occurred on average over a period of 24.5 min (±5.46 min SD, range 12.1–33.2 min). The mean height of the individuals in experiment 2a was 42.8 cm (±18.0 cm SD, range 8–73 cm). The mean number of leaves per plant was 74.5 (±52.6 SD, range 11–225). The mean leaf length was 42.5 mm (±14.1 mm SD, range 20.5–62.2 mm). In experiment 2b, the mean inter-trial time was 3 h 58 min (±26.3 SD min, range 2 h 55 min–4 h 48 min). The mean height of the individuals in experiment 2b was 40.3 cm (±11.8 cm SD, range 19.5–60.1 cm). The mean number of leaves per plant was 84.3 (±36.4 SD, range 22–157). The mean leaf length was 40.1 mm (±9.8 mm SD, range 28–61.1 mm).

**Figure 2:**
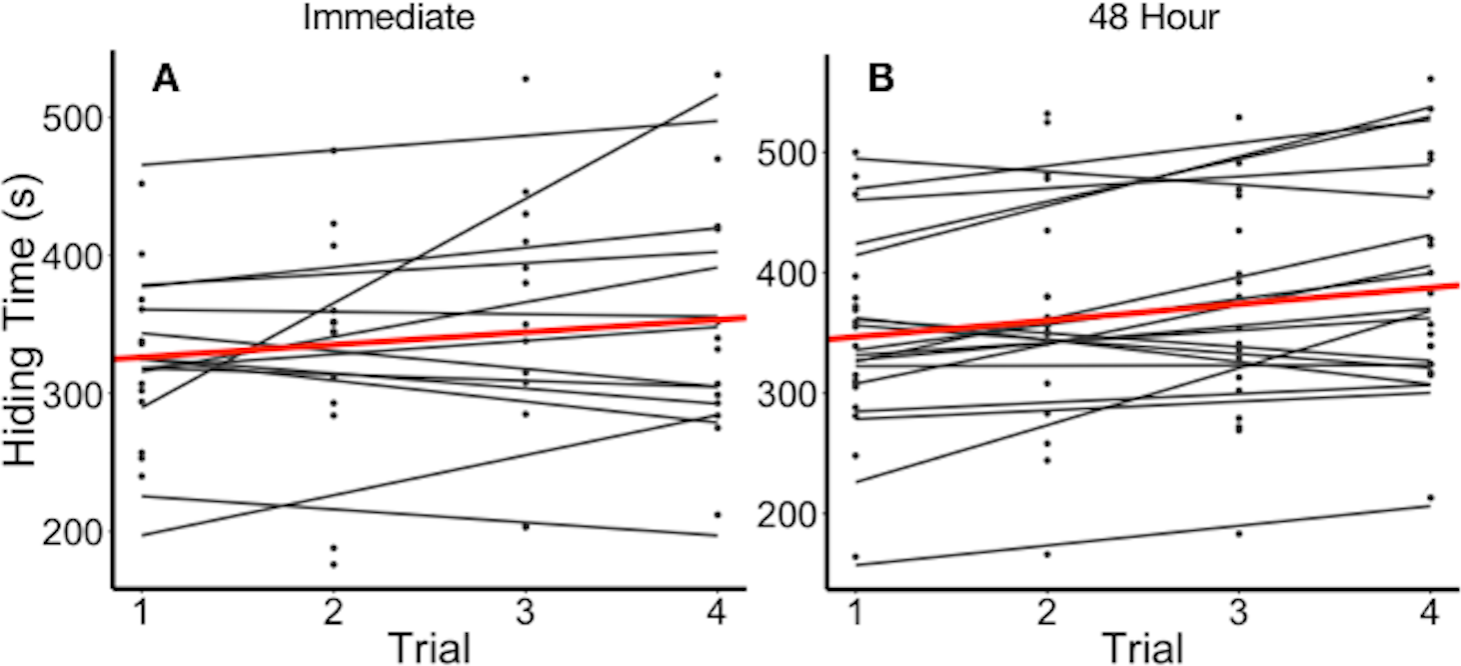
Hiding time (s) as a function of trial number, grouped by individual. (A) Data points and regression lines for individuals stimulated immediately upon reopening (experiment 2a). (B) Data points and regression lines for individuals stimulated every 48 h (experiment 2b). Thick line (red in on-line version) illustrates the mean hiding time across trials.

#### Results

Plants varied in their average hiding time (Fig. 2). In experiment 2a, we found that the full model including the fixed effects of log_10_(number of leaves), leaf length, and trial as parameters best explained variation in hiding time (Table 2). The full model with the above fixed effects was significantly better than either a null model (*p* < 2.2e^−16^), or a null model only with random effects of individual plant (*p* = 0.0001442). It was not significantly different from a full model that included estimates of the random slope with (*p* = 0.64). The full model showed that plants differed in their mean hiding time and the adjusted repeatability revealed that 75% of this variation was explained by the individual plant. The intraclass correlation coefficient (ICC) was 78.3%. Hiding time increased by 5.05 s (± 1.40 SEM) for each 1 mm increase in leaf length (*t*-value = 3.597). Hiding time also increased with the log_10_ number of leaves per plant (27.1 ± 43.5 SEM, *t*-value = 0.623). Hiding time increased with trial as well (13.6 ± 3.98 SEM, *t*-value = 3.42).

**Table 2:** A comparison of models fitted in R to explain changes in leaf hiding time in response to repeated immediate stimulation for experiment 2a. Data for hiding time were not transformed.

Description	Model	AIC
Null model	Hiding time ∼ 1	856
Null model with random effect only	Hiding time ∼1∣ID	797
Full model	Hiding time ∼ Leaf length + log_10_ Number of leaves + Trial + (1∣ID)	782^a^
Full model with random slope	Hiding time ∼ Leaf length + log_10_ Number of leaves + Trial + (1 + Trial∣ID)	785

**Notes.**

a LRT comparison to null model (*p* < 2.2e^−16^).

In experiment 2b, we found that the null model with the random effect of ID best explained variation in hiding time (Table 3). The random effects only model was significantly better fitting than a null model (*p* < 2.2e^−16^). It was not significantly better than a full model (*p* = 0.132), or a full model that included estimates of the random slope (*p* = 0.084). The null model with random effects only showed that plants differed in their average hiding time and the adjusted repeatability revealed that 65% of this variation was explained by individual plant. The ICC for this experiment was 63%.

**Table 3:** A comparison of models fitted in R to explain changes in leaf hiding time in response to stimulation every 48 h for experiment 2b. Data for hiding time were not transformed.

Description	Model	AIC
Null model	Hiding time ∼ 1	608
Null model with random effect only	Hiding time ∼1∣ID	584^a^
Full model	Hiding time ∼ Leaf length + Trial + (1∣ID)	584
Full model with random slope	Hiding time ∼ Leaf length + Trial + (1 + Trial∣ID)	583

**Notes.**

a LRT comparison to null model (*p* < 2.2e^−16^).

### Experiment 3: is variation in hiding time condition-dependent?

#### Methods

Experiment 2 showed that the percent of variation explained by individual declined as time increased. Conditions change more over a 48 h period than they do over a 24 min period. To examine the effect of condition on an individual’s behavior, we manipulated sunlight exposure and tested hiding time before and after treatment.

To determine how light availability affects sensitive plant hiding time, we exposed individual plants to one of three light treatments. We aimed to treat all individuals for 6 h, but the actual average treatment duration was 5.9 h (±0.33 h SD, range 5.17–7.82 h). These treatments were designed to deprive the plants of light and to control for any physical disturbance that may induce leaf closure. Prior to any manipulation, we measured the hiding time of a single leaf on each individual. Leaves were then marked with flagging tape for measurement post-treatment. After treatment, we waited 5–7 min for the plant to reopen, then re-measured the hiding time of the same marked leaf. We then deprived 20 sensitive plants of light by placing black, plastic HDX garbage bags (Atlanta, GA) over them (two plants died after this treatment and were thus eliminated from subsequent analysis). To control for the effect of disturbance caused by placing the bags on the plants, we also placed clear, plastic HDX garbage (Atlanta, GA) bags on 19 plants (one plant died after this treatment and was thus eliminated from subsequent analysis). The clear bags controlled for any disturbance caused by putting a bag on the plant, while allowing light to pass through. We acknowledge that the bags may have also manipulated the temperature and the humidity. However, this increase is likely to be similar between black and clear bags, which means the resulting effect is largely due to differences in sunlight availability. Our third treatment was a control treatment in which we recorded the hiding time of a leaf, marked it, and measured the hiding time again after the same interval (around 6 h) that we used for the two experimental manipulations. We performed this treatment on 20 individuals. In this experiment, the sample size was 56 individuals. Plants were randomly assigned to different treatments. To compare the effects of treatment, the model formally compares the slopes of individuals between trials, across treatments. The mean height of the individuals in experiment 3 was 40.0 cm (±21.5 cm SD, range 5.5–108.5 cm). The mean number of leaves per plant was 77.4 (±52.3 SD, range 16–279). The mean leaf length was 43.2 mm (±11.4 mm SD, range 18.9–63.3 mm).

#### Results

In experiment 3, we found that the full model that included the fixed effects of treatment and trial along with the interaction between treatment and trial best explained variation in hiding time (Table 4). The full model with random slope and the above fixed effects was significantly better than either a full model that included measurement as a parameter (*p* = 0.004), or a null random model (*p* = 0.00003).

**Table 4:** A comparison of models fitted in R to explain changes in leaf hiding time in response to treatment by covering in either a clear, black, or no bag as per Experiment 3. Data for hiding time were not transformed.

Description	Model	AIC
Null model	Hiding time ∼ 1	1,331
Null model with random effect only	Hiding time ∼1∣ID	1,330
Full model	Hiding time ∼ Measurement +1∣ID	1,315
Full model with random slope	Hiding time ∼ Measurement^a^ Treatment + (1 + trial∣ID)	1,308^a^

**Notes.**

a LRT comparison to null model (*p* < 2.2e^−16^).

**Figure 3:**
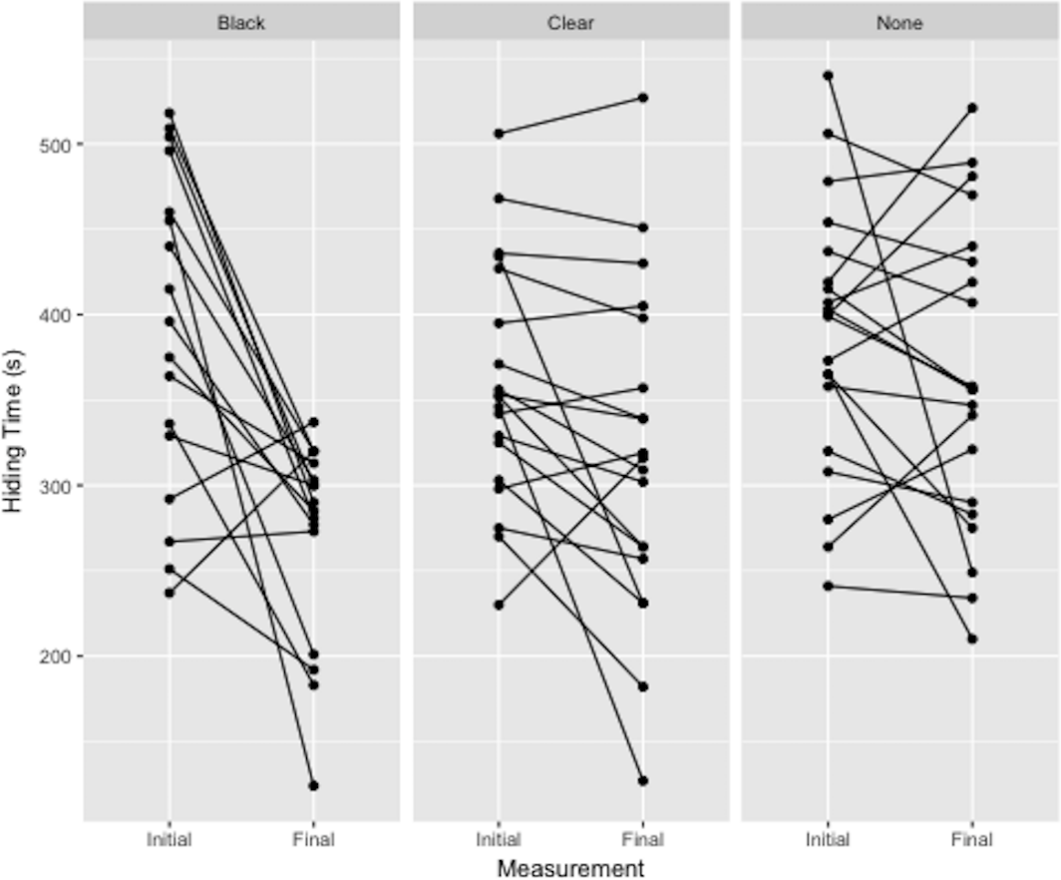
Difference in hiding time (s) as a function of total plant treatment (black plastic bag, clear plastic bag, no bag). Plotted values are the initial and final hiding times connected by a sloped line.

There was a significant effect of treatment on hiding time (Fig. 3). Plants deprived of photosynthesis with the black-bag treatments had significantly shorter hiding times post-treatment than did control (*p* = 0.002) or clear-bag treatments (*p* = 0.018), which were not significantly different from each other (*p* = 0.097). The adjusted repeatability showed that 41% of this variation can be attributed to individual plant and the ICC was 58.8%.

### Experiment 4: are consistent responses at the level of the whole plant or at the level of the individual leaf?

#### Methods

To determine if light effects are localized or systemic across the entire plant, we covered half of a plant’s leaves to prevent photosynthesis in the covered half and tested hiding time before and after treatment. Prior to treatment, we measured the hiding time of one leaf on two sides of the plant and designated a treatment and non-treatment side for each plant. These leaves were marked with flagging tape around their woody stem for future measurement. We partially covered 17 sensitive plants by wrapping the leaves on the treatment side with aluminium foil. To control for effects of disturbance, we wrapped leaves on the treatment side of 17 sensitive plants in clear plastic (Saran™, Oakland, CA, USA) so that light could still pass through. We had an additional control group in which 19 plants were not subject to any treatment on either side. Again, we acknowledge that there may be the possibility of temperature and humidity effects. However, this increase is likely to be similar between foil wrapped and saran wrapped leaves, which means the resulting effect is largely due to differences in sunlight availability. We removed these treatments after an average of 5.8 h (±0.43 h SD, range 4.18–6.65 h). After the leaves reopened, we re-measured the hiding time of the same marked leaf. In this experiment, the sample size was 53 leaves from 51 different plants that were randomly assigned to different treatments. As with experiment 3, to compare the effects of treatment, the model formally compares the slopes of individuals between trials, across treatments. The mean height of the individuals on experiment 4 was 37.1 cm (±16.9 cm SD, range 5.5–79.5 cm). The mean number of leaves was 62.8 (±44.7 SD, range 10–223). The mean leaf length was 40.7 mm (±11.8 mm SD, range 11.5–70.4 mm).

#### Results

In experiment 4, we found that the full model including the fixed effect of measurement, best explained variation in hiding time (Table 5). The full model with the above fixed effect was significantly better than either a null model (*p* < 2.2e^−16^), null random model (*p* = 0.0035) or a full model with random slope that included the fixed effects of measurement, treatment, and control or treatment (*p* = 0.0165).

**Table 5:** A comparison of models fitted in R to explain changes in leaf hiding time in response to treatment by covering a leaf in clear plastic wrap, foil, or no treatment relative to changes in a leaf that received no treatment in experiment 4. Data for hiding time were not transformed.

Description	Model	AIC
Null model	Hiding time ∼ 1	2,570
Null model with random effect only	Hiding time ∼1∣ID	2,446
Full model	Hiding time ∼ measurement +1∣ID	2,439^a^
Full model with random slope	Hiding time ∼ measurement^a^ Treatment^a^ ControlorTreat + (1 + trial∣ID)	2,438

**Notes.**

a LRT comparison to null model (*p* < 2.2e^−16^).

There was no significant effect of treatment on hiding time of the treated leaf. There was no significant effect of the foil treatment with final and initial hiding time and with control or treatment type (*p* = 0.2413) when compared to the base model, which included clear plastic wrap treatment. Furthermore, there was no significant effect of the control treatment with final and initial hiding time and control or treatment type (*p* = 0.9236) when compared to the base model. The adjusted repeatability showed that 71% of this variation in hiding time can be attributed to individual plant and the ICC for this experiment was 70.8%. The estimate for the effect of measurement on hiding time was 23.019 (±7.772 SEM, *t*-value = − 2.962).

## Discussion

Taken together, our results show that individual sensitive plants vary in consistent ways that could, in animals, be seen as reflecting variation in personality. Indeed, the intra-class correlation coefficients estimated in each of the experiments ranged from 55.8% to 78.3%. The adjusted repeatability values ranged from 41% to 75%. This means that a substantial proportion of the variation in sensitive plant hiding time was explained by the individual plant, not the treatment that we applied to the plants.

These results expand on those in a recent report by Simon, Hodson & Roitberg (2016), which was published while we were conducting our experiments and which we were unaware of until recently, in which the authors showed that the hiding time of sensitive plants grown under controlled conditions varied consistently over several weeks according to environment and plant condition. Our results build upon Simon, Hodson & Roitberg (2016) by showing that individual differences are consistent in the field. Furthermore, our results demonstrate that decision to open and close leaves occurs on a plant level. This is a future area of research that Simon, Hodson & Roitberg (2016) urged others to explore.

Current models of personality suggest that differences in state and condition drive life history trade-offs, which ultimately lead to variation in an individual’s behavior and personality (Stamps, 2007; Biro & Stamps, 2008; Wolf & Weissing, 2010). This understanding of behavior integrates behavioral models, such as optimal escape theory, into the framework of personality. For example, if an animal is deprived of food in a developmental stage, changes in physiology or the need for compensatory growth may change how that individual behaves in the future (Stamps, 2007).

On a more proximate level, energy reserves may impact both the ability and need to forage (Wolf & Weissing, 2010; Simon, Hodson & Roitberg, 2016). A hungrier animal may forage longer and accept greater risk while foraging than an animal that is not hungry (Morgan, 1988). In plants, light availability impacts photosynthetic need and may affect plant behavior in the same way that energy reserves affect animal behavior. Indeed, there have been some previous attempts to apply optimal hiding time models to plant behavior (Jensen, Dill & Cahill, 2011; Gagliano et al., 2014; Simon, Hodson & Roitberg, 2016).

A previous study by Jensen, Dill & Cahill (2011), which partially motivated our present study, found that hiding time in sensitive plants depended on light availability. They also reported great variability in hiding time between plants. Cahill Jr et al. (2012) speculated that this variability in hiding time was due to changes in environmental conditions, which is what we found (Fig. 3). Thus, we have shown that hiding time, an individually specific trait, is dependent upon light availability and, hence, plant condition.

Our final experiment showed that sensitive plant condition, rather than leaf condition, influenced hiding time. By contrast, Cahill et al. (2012) reported that sensitive plants behaved in a manner localized to each leaf and Simon, Hodson & Roitberg (2016) did not explore if decisions on whether to photosynthesize were made on a leaf or plant level. When Cahill Jr et al. (2012) damaged leaflets, the hiding time of that leaf increased, but surrounding leaves remained unaffected, indicating that response to damage is highly localized. This reaction may be limited to response to damage as we were unable to repeat these results when we manipulated light. When we deprived half a plant of light, there was no difference between the hiding time of covered and uncovered leaves. This suggests that leaves responded to a stimulus based on the condition of the whole plant rather than the condition of a single leaf. Because sensitive plants respond to light condition as a whole, we were able to apply behavioral models traditionally applied to animals.

While prior research on the degree of systemic or localized behavior in plants is limited, some studies’ experimental methods make certain assumptions about the degree to which responses are based on plant condition. Applewhite (1972) tested hiding time in multiple leaves from individual sensitive plants, but treated each leaf as a distinct individual by removing it from the plant for tests. This strategy assumes that leaf response is localized which is an assumption that our results clearly refute. Simon, Hodson & Roitberg (2016) also tested individual leaves, albeit attached to an entire plant, as an indicator of the behavior of the entire plant. By contrast, in order to examine differences in hiding time in plants reared under different light conditions, Gagliano et al. (2014) shook entire plants to close their leaves. This strategy assumes that plants respond to light condition and disturbance in a systemic manner. While we studied leaves, our experiments demonstrated that hiding time decisions are based on plant not leaf condition.

There has also been mixed evidence of whether leaves or plants habituate to repeated benign stimulation. Applewhite (1972) found that individual sensitive plants did not habituate if the leaves were not given time to reopen between stimulations, while Gagliano et al. (2014) concluded that habituation occurred only when leaves were exposed to continuous stimulation. We found no evidence of habituation in sensitive plants under the conditions we studied them. Individual leaf reopening time did not significantly differ over immediate or 48 h time intervals (Fig. 2). Although there has been no evidence of biochemical processes for habituation in the potassium-signalling mechanism, previous studies have speculated that the signalling network in sensitive plants is similar to that of animals, and could result in stress imprints which allow for habituation (Gagliano et al., 2014). However, our experiments were conducted at the scale of an individual leaf, rather than the entire plant. While habituation did not occur in our experiments, it is possible that habituation is dependent on the scale and the nature of the stimulus.

We found some evidence of sensitization; in experiment 2a hiding time increased with the number of trials. Furthermore, there was no significant difference between the abilities of the full model with random slope and the full model to explain variation in hiding time. While the full model with random slope suggests that individuals do not vary in their sensitization, this result does not rule out the presence of sensitization.

Overall, models developed to understand animal antipredator behavior were successfully applied to understand plant behavior. These models predict that physiological condition should influence the cost of hiding. Silvertown & Gordon (1989) argued that direct application of game theory and animal behavior models to the study of plant behavior was beneficial to the field. Willson & Burley (1983) used models of kin selection, sexual selection, and mate choice in animals to explain plant behavioral strategies. We examined plant decision-making in the context of optimal escape theory. Optimal escape theory predicts that animals should behave to optimize the trade-off between antipredator behavior and foraging (Cooper, 2015; Cooper & Blumstein, 2015). Our results are broadly consistent with these models. To successfully apply optimal escape theory to plants shows that these models are not limited to explaining animal behavior. Additionally, we have identified, for the first time under field conditions, consistent individual differences in plant behavioral responses to disturbance. Plants, it seems, may have personality.

##  Supplemental Information

10.7717/peerj.3598/supp-1 Data S1 Supplementary Materials Data associated with each experiment.

## Additional Information and Declarations

### Field Study Permissions

The following information was supplied relating to field study approvals (i.e., approving body and any reference numbers): Field experiments were approved by the Government of French Polynesia.

### Data Availability

The following information was supplied regarding data availability: The raw data are contained in Data S1.
