# Supplementary material for: Sensitive plant (Mimosa pudica) hiding time depends on individual and state
Source: PeerJ. 2017 Jul 31;5:e3598. doi: 10.7717/peerj.3598 (PMC5541926; doi:10.7717/peerj.3598)
Supplement: Data S1 — Data associated with each experiment. [file peerj-05-3598-s001.docx]

**Supplementary Tables**

**Table 1.** Data included in the analysis for experiment 1.

| ID | Leaf | Hiding Time (s) | log10 Hiding Time | Leaf Length (mm) |
| --- | --- | --- | --- | --- |
| 8 | 1 | 348 | 2.54 | 51.37 |
| 8 | 2 | 386 | 2.59 | 58.61 |
| 8 | 3 | 257 | 2.41 | 43.41 |
| 8 | 4 | 291 | 2.46 | 40.57 |
| 8 | 5 | 336 | 2.53 | 23.48 |
| 8 | 6 | 309 | 2.49 | 51.83 |
| 8 | 7 | 380 | 2.58 | 25.98 |
| 8 | 8 | 343 | 2.54 | 34.77 |
| 8 | 9 | 193 | 2.29 | 45 |
| 8 | 10 | 257 | 2.41 | 53.71 |
| 47 | 1 | 385 | 2.59 | 67.77 |
| 47 | 2 | 452 | 2.66 | 34.95 |
| 47 | 3 | 448 | 2.65 | 42.87 |
| 47 | 4 | 431 | 2.63 | 30.52 |
| 47 | 5 | 369 | 2.57 | 41.17 |
| 47 | 6 | 363 | 2.56 | 44.26 |
| 47 | 7 | 406 | 2.61 | 48.81 |
| 47 | 8 | 430 | 2.63 | 44.21 |
| 47 | 9 | 457 | 2.66 | 51.13 |
| 47 | 10 | 355 | 2.55 | 33.35 |
| 25 | 1 | 598 | 2.78 | 21.94 |
| 25 | 2 | 565 | 2.75 | 34.39 |
| 25 | 3 | 458 | 2.66 | 35.76 |
| 25 | 4 | 394 | 2.6 | 31.5 |
| 25 | 5 | 483 | 2.68 | 27.52 |
| 25 | 6 | 412 | 2.61 | 22.41 |
| 25 | 7 | 552 | 2.74 | 31.69 |
| 25 | 8 | 427 | 2.63 | 26.93 |
| 25 | 9 | 394 | 2.6 | 29.97 |
| 25 | 10 | 469 | 2.67 | 5.91 |
| 37 | 1 | 235 | 2.37 | 47.53 |
| 37 | 2 | 382 | 2.58 | 39.92 |
| 37 | 3 | 336 | 2.53 | 23.03 |
| 37 | 4 | 308 | 2.49 | 34.21 |
| 37 | 5 | 402 | 2.6 | 40.53 |
| 37 | 6 | 379 | 2.58 | 25.85 |
| 37 | 7 | 447 | 2.65 | 44.21 |
| 37 | 8 | 404 | 2.61 | 45.37 |
| 37 | 9 | 406 | 2.61 | 41.92 |
| 37 | 10 | 409 | 2.61 | 32.74 |
| 48 | 1 | 465 | 2.67 | 48.66 |
| 48 | 2 | 432 | 2.64 | 46.59 |
| 48 | 3 | 336 | 2.53 | 65.23 |
| 48 | 4 | 383 | 2.58 | 45.88 |
| 48 | 5 | 339 | 2.53 | 55.43 |
| 48 | 6 | 314 | 2.5 | 45.58 |
| 48 | 7 | 529 | 2.72 | 41.48 |
| 48 | 8 | 312 | 2.49 | 45.91 |
| 48 | 9 | 504 | 2.7 | 34.21 |
| 48 | 10 | 559 | 2.75 | 42.91 |
| 12 | 1 | 254 | 2.4 | 27.69 |
| 12 | 2 | 186 | 2.27 | 28.68 |
| 12 | 3 | 288 | 2.46 | 19.23 |
| 12 | 4 | 245 | 2.39 | 28.69 |
| 12 | 5 | 215 | 2.33 | 26.26 |
| 12 | 6 | 191 | 2.28 | 17.45 |
| 12 | 7 | 326 | 2.51 | 32.33 |
| 12 | 8 | 329 | 2.52 | 30.16 |
| 12 | 9 | 171 | 2.23 | 35.91 |
| 12 | 10 | 210 | 2.32 | 23.9 |
| 63 | 1 | 289 | 2.46 | 37.73 |
| 63 | 2 | 825 | 2.92 | 45.13 |
| 63 | 3 | 327 | 2.51 | 28.57 |
| 63 | 4 | 208 | 2.32 | 26.34 |
| 63 | 5 | 398 | 2.6 | 46.26 |
| 63 | 6 | 465 | 2.67 | 31.36 |
| 63 | 7 | 503 | 2.7 | 34.76 |
| 63 | 8 | 562 | 2.75 | 12.86 |
| 63 | 9 | 331 | 2.52 | 40.92 |
| 63 | 10 | 690 | 2.84 | 10.96 |
| 42 | 1 | 212 | 2.33 | 23.02 |
| 42 | 2 | 339 | 2.53 | 36.51 |
| 42 | 3 | 459 | 2.66 | 30.45 |
| 42 | 4 | 236 | 2.37 | 30.37 |
| 42 | 5 | 363 | 2.56 | 33.07 |
| 42 | 6 | 206 | 2.31 | 39.98 |
| 42 | 7 | 275 | 2.44 | 31.75 |
| 42 | 8 | 251 | 2.4 | 24.39 |
| 42 | 9 | 260 | 2.41 | 32.36 |
| 42 | 10 | 323 | 2.51 | 28 |
| 7 | 1 | 270 | 2.43 | 49.99 |
| 7 | 2 | 343 | 2.54 | 55.33 |
| 7 | 3 | 358 | 2.55 | 85.48 |
| 7 | 4 | 430 | 2.63 | 32.08 |
| 7 | 5 | 441 | 2.64 | 9.45 |
| 7 | 6 | 460 | 2.66 | 22.6 |
| 7 | 7 | 384 | 2.58 | 41.35 |
| 7 | 8 | 425 | 2.63 | 52.73 |
| 7 | 9 | 481 | 2.68 | 54.18 |
| 7 | 10 | 501 | 2.7 | 33.38 |
| 21 | 1 | 490 | 2.69 | 37.2 |
| 21 | 2 | 473 | 2.67 | 37.5 |
| 21 | 3 | 369 | 2.57 | 37.6 |
| 21 | 4 | 286 | 2.46 | 48.9 |
| 21 | 5 | 222 | 2.35 | 45.8 |
| 21 | 6 | 298 | 2.47 | 38.8 |
| 21 | 7 | 248 | 2.39 | 44.5 |
| 21 | 8 | 323 | 2.51 | 31.5 |
| 21 | 9 | 326 | 2.51 | 24.8 |
| 21 | 10 | 409 | 2.61 | 34 |
| 16 | 1 | 210 | 2.32 | 43 |
| 16 | 2 | 180 | 2.26 | 37 |
| 16 | 3 | 199 | 2.3 | 49.7 |
| 16 | 4 | 219 | 2.34 | 35.6 |
| 16 | 5 | 196 | 2.29 | 35.5 |
| 16 | 6 | 208 | 2.32 | 40.9 |
| 16 | 7 | 283 | 2.45 | 41 |
| 16 | 8 | 325 | 2.51 | 55.6 |
| 16 | 9 | 383 | 2.58 | 55.5 |
| 16 | 10 | 150 | 2.18 | 36.1 |
| 34 | 1 | 220 | 2.34 | 50 |
| 34 | 2 | 199 | 2.3 | 58.8 |
| 34 | 3 | 263 | 2.42 | 45 |
| 34 | 4 | 285 | 2.45 | 58.7 |
| 34 | 5 | 285 | 2.45 | 58.8 |
| 34 | 6 | 305 | 2.48 | 56.7 |
| 34 | 7 | 338 | 2.53 | 50.2 |
| 34 | 8 | 369 | 2.57 | 49.3 |
| 34 | 9 | 214 | 2.33 | 45.9 |
| 34 | 10 | 257 | 2.41 | 46.2 |
| 10 | 1 | 354 | 2.55 | 29.3 |
| 10 | 2 | 311 | 2.49 | 24.3 |
| 10 | 3 | 187 | 2.27 | 24.1 |
| 10 | 4 | 243 | 2.39 | 18.5 |
| 10 | 5 | 255 | 2.41 | 15.9 |
| 10 | 6 | 372 | 2.57 | 22.5 |
| 10 | 7 | 294 | 2.47 | 25.2 |
| 10 | 8 | 260 | 2.41 | 21.1 |
| 10 | 9 | 229 | 2.36 | 22.4 |
| 10 | 10 | 270 | 2.43 | 27.8 |
| 3 | 1 | 321 | 2.51 | 45.3 |
| 3 | 2 | 278 | 2.44 | 30.7 |
| 3 | 3 | 381 | 2.58 | 43.7 |
| 3 | 4 | 382 | 2.58 | 20.8 |
| 3 | 5 | 368 | 2.57 | 13 |
| 3 | 6 | 473 | 2.67 | 4.7 |
| 3 | 7 | 376 | 2.58 | 27.1 |
| 3 | 8 | 227 | 2.36 | 20.4 |
| 3 | 9 | 256 | 2.41 | 35.7 |
| 3 | 10 | 239 | 2.38 | 35.6 |

**Table 2.** Data included in the analysis for experiment 2a.

| ID | Trial | Leaf Length (mm) | Hiding Time (s) | N Leaves | log10 N Leaves |
| --- | --- | --- | --- | --- | --- |
| 75 | 1 | 41.47 | 359 | 100 | 2 |
| 75 | 2 | 41.47 | 342 | 100 | 2 |
| 75 | 3 | 41.47 | 329 | 100 | 2 |
| 75 | 4 | 41.47 | 324 | 100 | 2 |
| 67 | 1 | 28.1 | 248 | 13 | 1.11 |
| 67 | 2 | 28.1 | 244 | 13 | 1.11 |
| 67 | 3 | 28.1 | 313 | 13 | 1.11 |
| 67 | 4 | 28.1 | 383 | 13 | 1.11 |
| 78 | 1 | 41.39 | 465 | 60 | 1.78 |
| 78 | 2 | 41.39 | 532 | 60 | 1.78 |
| 78 | 3 | 41.39 | 435 | 60 | 1.78 |
| 78 | 4 | 41.39 | 561 | 60 | 1.78 |
| 79 | 1 | 41.5 | 355 | 36 | 1.56 |
| 79 | 2 | 41.5 | 258 | 36 | 1.56 |
| 79 | 3 | 41.5 | 354 | 36 | 1.56 |
| 79 | 4 | 41.5 | 324 | 36 | 1.56 |
| 80 | 1 | 50 | 500 | 56 | 1.75 |
| 80 | 2 | 50 | 478 | 56 | 1.75 |
| 80 | 3 | 50 | 469 | 56 | 1.75 |
| 80 | 4 | 50 | 467 | 56 | 1.75 |
| 62 | 1 | 48.33 | 288 | 39 | 1.59 |
| 62 | 2 | 48.33 | 283 | 39 | 1.59 |
| 62 | 3 | 48.33 | 269 | 39 | 1.59 |
| 62 | 4 | 48.33 | 317 | 39 | 1.59 |
| 2 | 1 | 33.5 | 372 | 110 | 2.04 |
| 2 | 2 | 33.5 | 357 | 110 | 2.04 |
| 2 | 3 | 33.5 | 272 | 110 | 2.04 |
| 2 | 4 | 33.5 | 339 | 110 | 2.04 |
| 6 | 1 | 32.5 | 281 | 26 | 1.41 |
| 6 | 2 | 32.5 | 308 | 26 | 1.41 |
| 6 | 3 | 32.5 | 279 | 26 | 1.41 |
| 6 | 4 | 32.5 | 315 | 26 | 1.41 |
| 28 | 1 | 45.2 | 310 | 30 | 1.48 |
| 28 | 2 | 45.2 | 380 | 30 | 1.48 |
| 28 | 3 | 45.2 | 341 | 30 | 1.48 |
| 28 | 4 | 45.2 | 357 | 30 | 1.48 |
| 30 | 1 | 20.5 | 308 | 118 | 2.07 |
| 30 | 2 | 20.5 | 349 | 118 | 2.07 |
| 30 | 3 | 20.5 | 399 | 118 | 2.07 |
| 30 | 4 | 20.5 | 339 | 118 | 2.07 |
| 40 | 1 | 39.8 | 369 | 109 | 2.04 |
| 40 | 2 | 39.8 | 356 | 109 | 2.04 |
| 40 | 3 | 39.8 | 302 | 109 | 2.04 |
| 40 | 4 | 39.8 | 349 | 109 | 2.04 |
| 66 | 1 | 45.9 | 480 | 126 | 2.1 |
| 66 | 2 | 45.9 | 435 | 126 | 2.1 |
| 66 | 3 | 45.9 | 491 | 126 | 2.1 |
| 66 | 4 | 45.9 | 494 | 126 | 2.1 |
| 49 | 1 | 46.7 | 339 | 116 | 2.06 |
| 49 | 2 | 46.7 | 351 | 116 | 2.06 |
| 49 | 3 | 46.7 | 380 | 116 | 2.06 |
| 49 | 4 | 46.7 | 400 | 116 | 2.06 |
| 58 | 1 | 47.6 | 305 | 39 | 1.59 |
| 58 | 2 | 47.6 | 363 | 39 | 1.59 |
| 58 | 3 | 47.6 | 336 | 39 | 1.59 |
| 58 | 4 | 47.6 | 423 | 39 | 1.59 |
| 59 | 1 | 60.75 | 379 | 83 | 1.92 |
| 59 | 2 | 60.75 | 525 | 83 | 1.92 |
| 59 | 3 | 60.75 | 464 | 83 | 1.92 |
| 59 | 4 | 60.75 | 536 | 83 | 1.92 |
| 72 | 1 | 62.2 | 397 | 44 | 1.64 |
| 72 | 2 | 62.2 | 482 | 44 | 1.64 |
| 72 | 3 | 62.2 | 529 | 44 | 1.64 |
| 72 | 4 | 62.2 | 499 | 44 | 1.64 |
| 73 | 1 | 51.5 | 315 | 11 | 1.04 |
| 73 | 2 | 51.5 | 380 | 11 | 1.04 |
| 73 | 3 | 51.5 | 392 | 11 | 1.04 |
| 73 | 4 | 51.5 | 428 | 11 | 1.04 |
| 26 | 1 | 27.7 | 164 | 225 | 2.35 |
| 26 | 2 | 27.7 | 166 | 225 | 2.35 |
| 26 | 3 | 27.7 | 183 | 225 | 2.35 |
| 26 | 4 | 27.7 | 213 | 225 | 2.35 |

**Table 3.** Data included in the analysis for experiment 2b.

| ID | Trial | Leaf Length (mm) | Hiding Time (s) | N Leaves | log10 N Leaves |
| --- | --- | --- | --- | --- | --- |
| 48 | 1 | 61.11 | 368 | 74 | 1.87 |
| 48 | 2 | 61.11 | 284 | 74 | 1.87 |
| 48 | 3 | 61.11 | 338 | 74 | 1.87 |
| 48 | 4 | 61.11 | 307 | 74 | 1.87 |
| 47 | 1 | 44.2 | 452 | 118 | 2.07 |
| 47 | 2 | 44.2 | 476 | 118 | 2.07 |
| 47 | 3 | 44.2 | 528 | 118 | 2.07 |
| 47 | 4 | 44.2 | 470 | 118 | 2.07 |
| 8 | 1 | 42.59 | 294 | 52 | 1.72 |
| 8 | 2 | 42.59 | 351 | 52 | 1.72 |
| 8 | 3 | 42.59 | 308 | 52 | 1.72 |
| 8 | 4 | 42.59 | 293 | 52 | 1.72 |
| 7 | 1 | 50.28 | 361 | 74 | 1.87 |
| 7 | 2 | 50.28 | 423 | 74 | 1.87 |
| 7 | 3 | 50.28 | 391 | 74 | 1.87 |
| 7 | 4 | 50.28 | 419 | 74 | 1.87 |
| 3 | 1 | 48.61 | 302 | 22 | 1.34 |
| 3 | 2 | 48.61 | 344 | 22 | 1.34 |
| 3 | 3 | 48.61 | 315 | 22 | 1.34 |
| 3 | 4 | 48.61 | 275 | 22 | 1.34 |
| 10 | 1 | 28 | 240 | 45 | 1.65 |
| 10 | 2 | 28 | 188 | 45 | 1.65 |
| 10 | 3 | 28 | 203 | 45 | 1.65 |
| 10 | 4 | 28 | 332 | 45 | 1.65 |
| 12 | 1 | 26.67 | 326 | 48 | 1.68 |
| 12 | 2 | 26.67 | 312 | 48 | 1.68 |
| 12 | 3 | 26.67 | 285 | 48 | 1.68 |
| 12 | 4 | 26.67 | 284 | 48 | 1.68 |
| 16 | 1 | 41.19 | 338 | 120 | 2.08 |
| 16 | 2 | 41.19 | 293 | 120 | 2.08 |
| 16 | 3 | 41.19 | 350 | 120 | 2.08 |
| 16 | 4 | 41.19 | 352 | 120 | 2.08 |
| 21 | 1 | 32.84 | 307 | 157 | 2.2 |
| 21 | 2 | 32.84 | 345 | 157 | 2.2 |
| 21 | 3 | 32.84 | 430 | 157 | 2.2 |
| 21 | 4 | 32.84 | 531 | 157 | 2.2 |
| 25 | 1 | 29.04 | 257 | 103 | 2.01 |
| 25 | 2 | 29.04 | 407 | 103 | 2.01 |
| 25 | 3 | 29.04 | 410 | 103 | 2.01 |
| 25 | 4 | 29.04 | 340 | 103 | 2.01 |
| 34 | 1 | 44.99 | 401 | 87 | 1.94 |
| 34 | 2 | 44.99 | 360 | 87 | 1.94 |
| 34 | 3 | 44.99 | 380 | 87 | 1.94 |
| 34 | 4 | 44.99 | 421 | 87 | 1.94 |
| 37 | 1 | 41.07 | 336 | 75 | 1.88 |
| 37 | 2 | 41.07 | 352 | 75 | 1.88 |
| 37 | 3 | 41.07 | 446 | 75 | 1.88 |
| 37 | 4 | 41.07 | 299 | 75 | 1.88 |
| 42 | 1 | 30.71 | 253 | 121 | 2.08 |
| 42 | 2 | 30.71 | 176 | 121 | 2.08 |
| 42 | 3 | 30.71 | 204 | 121 | 2.08 |
| 42 | 4 | 30.71 | 212 | 121 | 2.08 |

**Table 4.** Data included in the analysis for experiment 3.

| ID | Treatment | Initial Hiding Time (s) | Final Hiding Time (s) | Difference in HT (s) | Treatment Duration (h) |
| --- | --- | --- | --- | --- | --- |
| 40 | Black | 496 | 281 | -215 | 6:06 |
| 45 | Clear | 303 | 231 | -72 | 6:00 |
| 44 | None | 373 | 419 | 46 | 6:00 |
| 39 | None | 400 | 481 | 81 | 6:00 |
| 35 | Black | 455 | 124 | -331 | 6:00 |
| 36 | Clear | 329 | 302 | -27 | 5:59 |
| 4 | Black | 336 | 183 | -153 | 6:00 |
| 5 | None | 280 | 321 | 41 | 5:56 |
| 6 | Clear | 371 | 339 | -32 | 6:00 |
| 49 | Black | 415 | 201 | -214 | 6:00 |
| 52 | None | 399 | 358 | -41 | 5:59 |
| 54 | Clear | 353 | 339 | -14 | 6:00 |
| 59 | Black | 364 | 313 | -51 | 6:00 |
| 58 | Clear | 395 | 405 | 10 | 5:59 |
| 60 | None | 415 | 356 | -59 | 5:58 |
| 24 | Black | 504 | 300 | -204 | 7:49 |
| 26 | Clear | 325 | 264 | -61 | 6:03 |
| 23 | None | 320 | 283 | -37 | 5:57 |
| 11 | Black | 518 | 290 | -228 | 6:00 |
| 2 | Clear | 270 | 182 | -88 | 5:35 |
| 33 | None | 540 | 249 | -291 | 5:39 |
| 31 | Black | 237 | 320 | 83 | 5:38 |
| 29 | Clear | 346 | 127 | -219 | 5:39 |
| 13 | None | 358 | 347 | -11 | 5:38 |
| 32 | None | 308 | 290 | -18 | 5:24 |
| 30 | Black | 460 | 320 | -140 | 5:21 |
| 28 | Clear | 352 | 264 | -88 | 5:20 |
| 46 | Black | 440 | 303 | -137 | 6:00 |
| 50 | Clear | 427 | 398 | -29 | 6:00 |
| 51 | None | 437 | 407 | -30 | 6:00 |
| 53 | None | 403 | 356 | -47 | 6:00 |
| 56 | Black | 251 | 192 | -59 | 6:00 |
| 61 | Clear | 356 | 309 | -47 | 6:00 |
| 62 | None | 241 | 234 | -7 | 6:00 |
| 65 | Black | 375 | 285 | -90 | 6:00 |
| 64 | Clear | 275 | 257 | -18 | 6:00 |
| 66 | None | 419 | 521 | 102 | 6:00 |
| 41 | Clear | 434 | 231 | -203 | 5:16 |
| 1 | Black | 396 | 277 | -119 | 6:02 |
| 9 | Clear | 230 | 316 | 86 | 5:56 |
| 14 | None | 365 | 210 | -155 | 5:58 |
| 15 | Black | 292 | 337 | 45 | 5:51 |
| 17 | Clear | 342 | 357 | 15 | 5:48 |
| 18 | None | 264 | 341 | 77 | 5:43 |
| 19 | Black | 267 | 273 | 6 | 5:55 |
| 20 | Clear | 298 | 319 | 21 | 5:39 |
| 22 | None | 365 | 275 | -90 | 5:41 |
| 27 | Black | 329 | 300 | -29 | 5:10 |
| 79 | Black | 509 | 320 | -189 | 6:20 |
| 78 | Clear | 506 | 527 | 21 | 6:14 |
| 72 | Clear | 436 | 430 | -6 | 5:59 |
| 77 | Clear | 468 | 451 | -17 | 5:54 |
| 69 | None | 478 | 489 | 11 | 5:51 |
| 80 | None | 454 | 431 | -23 | 5:51 |
| 70 | None | 506 | 470 | -36 | 5:49 |
| 75 | None | 407 | 440 | 33 | 5:48 |

**Table 5.** Data used in the analysis for experiment 4.

| ID | Treatment | Initial Hiding Time (T) | Initial Hiding Time (NT) | Final Hiding Time (T) | Final Hiding Time (NT) | DifferenceHT (T) | DifferenceHT (NT) |
| --- | --- | --- | --- | --- | --- | --- | --- |
| 5 | Foil | 310 | 195 | 290 | 268 | -20 | 73 |
| 36 | Clear | 282 | 191 | 205 | 158 | -77 | -33 |
| 44 | Foil | 272 | 186 | 163 | 299 | -109 | 113 |
| 45 | Clear | 247 | 352 | 295 | 317 | 48 | -35 |
| 52 | Foil | 285 | 235 | 145 | 169 | -140 | -66 |
| 39 | Clear | 368 | 307 | 316 | 282 | -52 | -25 |
| 4 | Clear | 350 | 328 | 262 | 189 | -88 | -139 |
| 6 | None | 309 | 294 | 340 | 335 | 31 | 41 |
| 36 | Clear | 334 | 357 | 308 | 351 | -26 | -6 |
| 40 | None | 367 | 327 | 290 | 298 | -77 | -29 |
| 45 | Clear | 240 | 311 | 262 | 258 | 22 | -53 |
| 49 | None | 266 | 335 | 270 | 283 | 4 | -52 |
| 54 | Clear | 260 | 389 | 198 | 344 | -62 | -45 |
| 59 | None | 368 | 313 | 302 | 295 | -66 | -18 |
| 24 | Foil | 301 | 292 | 161 | 288 | -140 | -4 |
| 11 | Foil | 231 | 189 | 386 | 206 | 155 | 17 |
| 31 | Foil | 193 | 207 | 92 | 149 | -101 | -58 |
| 29 | Foil | 135 | 185 | 144 | 158 | 9 | -27 |
| 58 | None | 252 | 284 | 303 | 340 | 51 | 56 |
| 66 | None | 384 | 479 | 405 | 483 | 21 | 4 |
| 34 | Clear | 385 | 323 | 290 | 293 | -95 | -30 |
| 37 | None | 328 | 396 | 386 | 364 | 58 | -32 |
| 42 | None | 383 | 366 | 320 | 316 | -63 | -50 |
| 41 | Clear | 409 | 358 | 356 | 360 | -53 | 2 |
| 46 | Clear | 275 | 287 | 252 | 269 | -23 | -18 |
| 47 | Clear | 316 | 253 | 284 | 246 | -32 | -7 |
| 50 | Clear | 307 | 249 | 327 | 292 | 20 | 43 |
| 56 | None | 260 | 218 | 289 | 255 | 29 | 37 |
| 3 | Foil | 207 | 317 | 122 | 236 | -85 | -81 |
| 7 | Foil | 251 | 268 | 107 | 265 | -144 | -3 |
| 10 | None | 270 | 282 | 191 | 219 | -79 | -63 |
| 8 | Foil | 201 | 186 | 80 | 199 | -121 | 13 |
| 12 | Foil | 142 | 235 | 97 | 390 | -45 | 155 |
| 5 | Foil | 246 | 193 | 148 | 172 | -98 | -21 |
| 21 | None | 224 | 325 | 206 | 345 | -18 | 20 |
| 16 | None | 139 | 223 | 178 | 109 | 39 | -114 |
| 90 | None | 742 | 623 | 666 | 571 | -76 | -52 |
| 97 | Clear | 483 | 480 | 515 | 492 | 32 | 12 |
| 86 | Clear | 406 | 482 | 401 | 522 | -5 | 40 |
| 93 | Clear | 436 | 486 | 500 | 484 | 64 | -2 |
| 85 | Clear | 454 | 429 | 342 | 341 | -112 | -88 |
| 91 | Clear | 388 | 342 | 391 | 312 | 3 | -30 |
| 94 | Clear | 368 | 367 | 293 | 297 | -75 | -70 |
| 98 | None | 379 | 328 | 318 | 278 | -61 | -50 |
| 92 | Foil | 392 | 276 | 284 | 111 | -108 | -165 |
| 84 | Foil | 280 | 220 | 305 | 326 | 25 | 106 |
| 88 | None | 343 | 361 | 223 | 260 | -120 | -101 |
| 87 | Foil | 213 | 238 | 124 | 159 | -89 | -79 |
| 89 | None | 268 | 234 | 139 | 205 | -129 | -29 |
| 81 | Foil | 249 | 241 | 288 | 217 | 39 | -24 |
| 83 | Foil | 371 | 306 | 446 | 260 | 75 | -46 |
| 95 | None | 333 | 316 | 389 | 349 | 56 | 33 |
| 96 | None | 298 | 279 | 426 | 453 | 128 | 174 |
